# A Manual of Dental Mechanics

**Published:** 1873-07

**Authors:** 


*A Manual of Dental Mechanics.*—By Oakley Coales, L. R C. S. Publishers, Lindsay & Blakiston, Philadelphia.

This is the first edition of another English work, containing two hundred and eighty-three pages, and one hundred and forty illustrations, printed in large type and on heavy paper. The author in his pr eface states that this work makes no pretence to originality, but is intended for the student rather than the practitioner. The information it imparts is of a practical nature, relating to dental mechanics, and excluding all that is of doubtful value or merely theoretical. To his American brethren the author expresses his deep obligations for valuable investigations and new facts in the Science of Dentistry.

Section I. treats of the Preparation of the Mouth for Artificial Teeth. Section II. On Taking Impressions. Section III. Various Modes of Applying Heat in the Laboratory. Section IV. Casting in Plaster of Paris and Metal. Section V. Precious Metals used in Dentistry. Section VI. Making Gold Plates. Section VII. Various Forms of Porcelain used in Mechanical Dentistry. Section VIII. Pivot Teeth. Section IX. Choosing and Adjusting Mineral Teeth. Section X. The Vulcanite Base. Section XI. The Celluloid Base. Section XII. Treatment of Deformities of the Mouth. Appendix, containing receipts for making gold plate and solder.

